# Serological and Histological Examination of a Nonalcoholic Steatohepatitis Mouse Model Created via the Administration of Monosodium Glutamate

**DOI:** 10.1155/2014/725351

**Published:** 2014-10-30

**Authors:** Atsuko Takai, Kentaro Kikuchi, Yusuke Kajiyama, Anna Sugiura, Masatsugu Negishi, Hiromichi Tsunashima, Hanae Yamada, Kotaro Matsumoto, Koichi Tsuneyama, Yuki Moritoki, Masumi Hara, Hiroshi Miyakawa

**Affiliations:** ^1^Fourth Department of Internal Medicine, Teikyo University Mizonokuchi Hospital, 3-8-3 Mizonokuchi, Takatsu-ku, Kawasaki-shi, Kanagawa 213-8507, Japan; ^2^Department of Diagnostic Pathology, Graduate School of Medicine and Pharmaceutical Science, University of Toyama, 2630 Sugitani, Toyama-shi, Toyama 930-0194, Japan; ^3^Department of General Medical Practice and Laboratory Diagnostic Medicine, Akita University Graduate School of Medicine, 1-1-1 Hondo, Akita-shi, Akita 010-8543, Japan

## Abstract

The administration of monosodium glutamate (MSG) to mice induces hepatic steatosis and inflammation. In this study, we investigated the metabolic features of MSG-treated mice and the histological changes that occur in their livers and adipose tissue. MSG mice were prepared by subcutaneously injecting MSG into newborn C57BL/6J male mice. The control mice were subcutaneously injected with saline. Another group of mice was fed a methionine- and choline-deficient diet (MCD). Compared with the control mice, the MSG mice had higher serum levels of insulin and cholesterol than the control mice, whereas the opposite was true for the MCD mice. Microvesicular steatosis and inflammatory cell infiltration were detected in both the MSG and MCD mouse livers. Enlarged adipocytes and crown-like structures were observed in the epididymal fat of the MSG mice, whereas neither of these features was seen in the MCD mice. Flow cytometric analysis revealed increased frequencies of monocytes and M1 macrophages in the livers and epididymal fat tissue of the MSG mice, respectively. The MSG mice exhibited the characteristic liver histopathology of nonalcoholic steatohepatitis (NASH) as well as metabolic syndrome-like features, which suggested that MSG mice are a better model of human NASH than MCD mice.

## 1. Introduction

Nonalcoholic fatty liver disease (NAFLD) and nonalcoholic steatohepatitis (NASH) are hepatic phenotypes of metabolic syndrome. These conditions start as fatty liver and eventually progress to liver cirrhosis and cancer in association with insulin resistance and so-called second hits such as oxidative stress and inflammatory cytokine production [1]. In Japan, NAFLD afflicts 1 in 3 adults [2] and NASH that advances to liver cirrhosis exhibits a cumulative 5-year cancer incidence rate of 20% [3]. Thus, it is important to elucidate the pathogenesis of NASH. Research using mouse models is essential for achieving this, but not all existing NAFLD/NASH mouse models display metabolic syndrome-like features [4].

Subcutaneously injected monosodium glutamate (MSG) damages the pathway from the arcuate nucleus of the hypothalamus to the paraventricular nuclei, resulting in obesity [5, 6] as well as fatty liver, inflammatory cell infiltration, and fibrosis [7–11]. Thus, MSG-treated mice (MSG mice) might be a useful model of human NAFLD/NASH. However, there have been few reports on the blood glucose and serum lipid levels of MSG mice or their metabolic parameters. In this study, we evaluated the hepatic histopathology of C57BL/6J mice that had been administered MSG as well as the amount of visceral fat that they possessed and their blood glucose and serum lipid levels to assess the potential value of MSG mice as a model of human NAFLD/NASH.

## 2. Materials and Methods

### 2.1. Creating the NAFLD/NASH Model Mice

We created the MSG-induced mouse model of NAFLD/NASH by subcutaneously injecting MSG into C57BL/6J mice, as described previously [7]. MSG (Wako Pure Chemical Industries Ltd., Osaka, Japan) in normal saline was subcutaneously injected at a dose of 4 mg/g body weight into the backs of 6 male C57BL/6J mice (Charles River Laboratories Japan Inc., Kanagawa, Japan) within 5 days of their birth using a 30 G needle. The animals were then housed under standard dietary and environmental conditions. As a control group, 6 age-matched C57BL/6J males were subcutaneously injected with the same volume of normal saline and housed under identical conditions. Another group of 6 male C57BL/6J mice were raised on a normal diet up to 6 weeks of age and then switched to a methionine- and choline-deficient (MCD) diet to establish an MCD-induced model of NAFLD/NASH. This study was conducted with the approval of the Teikyo University Committee on Laboratory Animals and the Ethical Committee on Laboratory Animals (Teikyo Medical Animals 09-009) and was implemented in accordance with institutional guidelines.

### 2.2. Histological and Serological Analyses

We measured the body weight and dietary intake of each mouse periodically up to 18 weeks of age, at which point their fasting blood glucose, serum insulin, total serum cholesterol, and serum alanine transaminase (ALT) levels were measured after 10-hour fasting, and then the mice were sacrificed by cervical dislocation. The liver and epididymal fat of the mice was excised and weighed, and some tissue samples were fixed with formalin and stained with hematoxylin-eosin for histological evaluation. The liver samples were evaluated pathologically using the NAFLD activity score (NAS), and the remaining tissues were used for flow cytometry.

### 2.3. Isolation of Mononuclear Cells from Liver and Epididymal Adipose Tissue

After perfusing the liver of each mouse with phosphate-buffered saline (PBS) containing 0.5% bovine serum albumin and 0.04% ethylenediaminetetraacetic acid, the liver cells were dissociated by passing the tissue through a cell strainer with a mesh size of 40 *μ*m (BD Falcon, Durham, NC, USA). The cells were then suspended in PBS and centrifuged for 5 minutes at 500 rpm. The supernatant was centrifuged for 5 minutes at 1200 rpm to precipitate hepatic cells. The resultant precipitate was resuspended in PBS buffer, layered on Lymphoprep (Axis-Shield Proc. AS, Oslo, Norway) with a relative density of 1.077, and centrifuged for 15 minutes at 1400 rpm to yield the hepatic mononuclear cell (HMNC) fraction.

Epididymal adipose tissue was excised from the peritoneal cavity of each mouse, before being thinly sliced, incubated in Hanks buffer containing 0.05% collagenase A at 37°C for 45 minutes, and dissociated by passing it through a cell strainer. The dissociated cells were resuspended in PBS buffer and centrifuged for 5 minutes at 1000 rpm. The supernatant was discarded, and the precipitate was resuspended in PBS buffer, layered on Lymphoprep, and centrifuged for 15 minutes at 1400 rpm to yield a stromal vascular fraction (SVF). Each layer was collected with a pipette and washed in PBS buffer, and then the number of viable cells was counted using trypan blue staining.

### 2.4. Flow Cytometry

For cell surface staining, 1 *μ*L of purified anti-mouse CD16/32 antibodies in 24 *μ*L of cell staining buffer (formulated to prevent nonspecific staining) was added to 1 × 10^6^ HMNC or SVF cells from three groups of six mice in a 1.5 mL tube, and the mixture was incubated at 4°C for 10 minutes. All samples were triplicated. Phycoerythrin- (PE-) labeled anti-mouse F4/80 antibodies, allophycocyanin (APC)/Alexa Fluor 750-labeled anti-mouse CD11b antibodies, and APC-labeled anti-mouse CD115 antibodies in 25 *μ*L of cell staining buffer were added and allowed to react in darkness at 4°C for 15 minutes to identify the mononuclear leukocytes in the HMNC suspension. Alexa Fluor 488-labeled anti-mouse CD206 antibodies, PE-labeled anti-mouse F4/80 antibodies, and APC-labeled anti-mouse CD11c antibodies were added to the SVF, and the resultant mixture was incubated under the same conditions. All of the antibodies and the cell staining buffer were from BioLegend (San Diego, CA, USA). After being washed, the labeled cells were resuspended in 200 *μ*L of PBS, transferred into the wells of 96-well round-bottomed plates, and analyzed on a BD FACSArray flow cytometer using the software FACSArray (BD Immunocytometry Systems, San Jose, CA, USA).

### 2.5. Statistical Analysis

Mouse body weight, liver weight, epididymal adipose tissue weight, blood glucose, serum insulin, total serum cholesterol, serum ALT, and the ratios of monocytes and macrophages are expressed as mean ± standard error of the mean (SEM) values. All statistical analyses were performed using the nonparametric Mann-Whitney test and StatView version 5.0 for Macintosh (SAS institute Inc., Cary, NC, USA). *P* < 0.05 was used as the criterion for statistical significance.

## 3. Results

### 3.1. Time Course of the Body Weight Changes Seen in the MSG Mice

**Figure 1:**
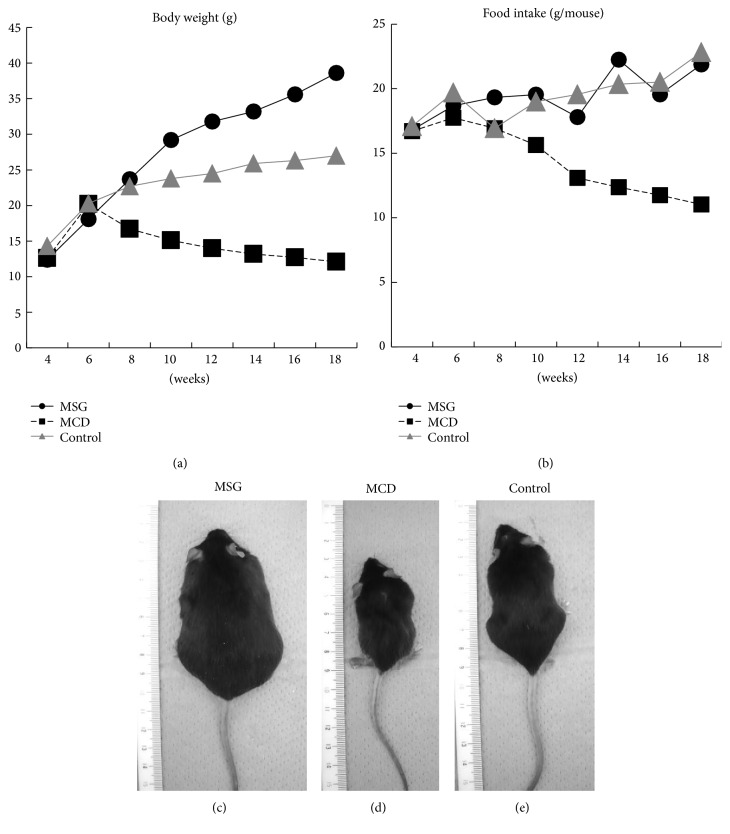
Changes in body weight and dietary intake and photographs of the MSG, MCD, and control mice obtained at 18 weeks of age. The mean body weight of each group (*n* = 6 mice/group) and the mean weekly dietary intake per mouse are shown. MSG: mice that were subcutaneously injected with monosodium glutamate; MCD: mice fed a methionine- and choline-deficient diet; control: control mice.

At 6 weeks of age, all 3 groups of mice exhibited similar mean body weights (MSG: 19.0 ± 0.9 g, MCD: 21.2 ± 0.6 g, and control: 20.8 ± 0.4 g). However, the MSG mice displayed heavier body weights than the control mice from 10 weeks of age (Figure 1). Although their dietary intake did not differ, the MSG mice were significantly heavier than the control mice at 18 weeks (38.9 ± 1.7 g versus 26.0 ± 1.0 g; *P* < 0.001). On the other hand, the body weights and dietary intake of the MCD mice started declining after the dietary switch. During the 17th and 18th weeks, the mean dietary intake of the MCD mice was 11.6 g. On the other hand, that of the controls was 22.1 g. The mean body weight of the MCD mice reached 12.7 ± 0.6 g at 18 weeks of age.

### 3.2. Liver and Visceral Fat Weight and Serological Characteristics

**Figure 2:**
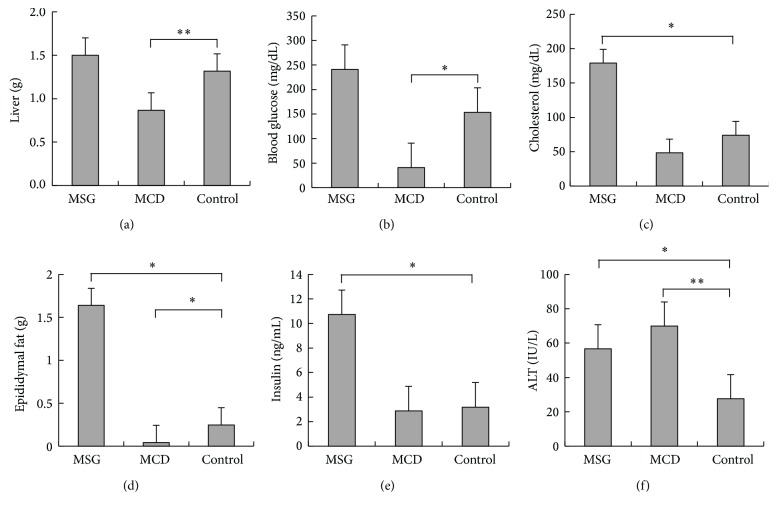
Comparisons of the liver and epididymal adipose tissue weight and fasting blood glucose, serum insulin, total cholesterol, and ALT levels of the mice at 18 weeks of age. MSG: mice that were subcutaneously injected with monosodium glutamate; MCD: mice fed a methionine- and choline-deficient diet; control: control mice. Mean ± SEM values for each group are shown. ^*^ *P* < 0.05; ^**^ *P* < 0.01.

The liver weight of the MSG mice was greater than that of the control mice (1.5 ± 0.3 g versus 1.3 ± 0.2 g), but the difference did not reach statistical significance (Figure 2). The epididymal adipose tissue weight of the MSG mice was significantly higher than that of the control mice (1.6 ± 0.2 g versus 0.2 ± 0.1 g; *P* = 0.01). Conversely, both the liver weight (0.9 ± 0.2 g, *P* = 0.005) and epididymal adipose tissue weight (0.04 ± 0.03 g, *P* = 0.02) of the MCD mice were significantly lower than those of the control mice.

The MSG mice exhibited a higher fasting blood glucose level than the control mice, but the difference was not statistically significant (241 ± 55 mg/dL versus 153.5 ± 56.3 mg/dL). The mean insulin level of the MSG mice was significantly higher than that of the control mice (10.7 ± 3.8 ng/mL versus 3.2 ± 1.7 ng/mL; *P* = 0.03). The total cholesterol level of the MSG mice was also significantly elevated compared with that of the control mice (179 ± 31.7 mg/dL versus 74.1 ± 8.8 mg/dL; *P* = 0.02). On the other hand, both the blood glucose (41 ± 32.5 mg/dL, *P* = 0.006) and total cholesterol levels (48.4 ± 19.9 mg/dL, *P* = 0.19) of the MCD mice were lower than those of the control mice, and there were no signs of hyperinsulinemia (2.8 ± 1.5 ng/mL, *P* = 0.28). Both the MSG and MCD mice displayed significantly higher serum ALT levels (56.7 ± 8.6 IU/L and 70.1 ± 8.7 IU/L, resp.) than the control mice (27.7 ± 7.8 IU/L; *P* = 0.01).

### 3.3. Histopathological Characteristics of the Liver and Visceral Fat

**Figure 3:**
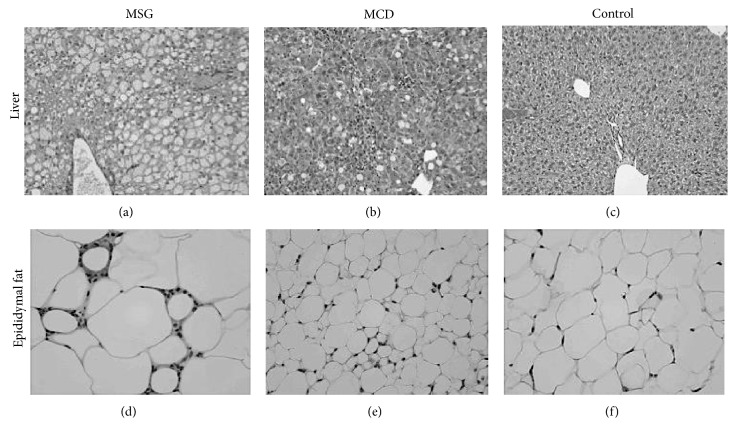
Hematoxylin-eosin staining (400× magnification) of the liver and epididymal adipose tissue at 18 weeks. Photographs of one representative animal from each group are shown. Lipid accumulation and ballooning in the liver, increased amounts of visceral fat, and crown-like structures (arrow) were noted in the MSG mice. Data for one representative mouse per group are shown. MSG: mice that were subcutaneously injected with monosodium glutamate; MCD: mice fed a methionine- and choline-deficient diet; control: control mice.

The livers of the MSG mice were characterized by swollen hepatocytes, microvesicular steatosis, and inflammatory cell infiltration (Figure 3). Similarly, fat droplets and marked inflammatory cell infiltration were observed in the livers of the MCD mice. The inflammation scores of the MCD mice were significantly higher than those of the MSG mice (2.6 ± 0.4 versus 1.5 ± 0.5, *P* = 0.006), whereas hepatocellular ballooning was significantly severer in the MSG mice than in the MCD mice (2.0 ± 0.0 versus 1.5 ± 0.5, *P* = 0.03). Enlarged adipocytes and crown-like structures were observed in the epididymal fat tissue of the MSG mice, whereas atrophied adipocytes were seen in the epididymal fat tissue of the MCD mice, which was consistent with their weight loss.

### 3.4. Frequencies of Monocytes in the Hepatic Mononuclear Cell Fraction and Macrophages in the Epididymal Stromal Vascular Fraction

**Figure 4:**
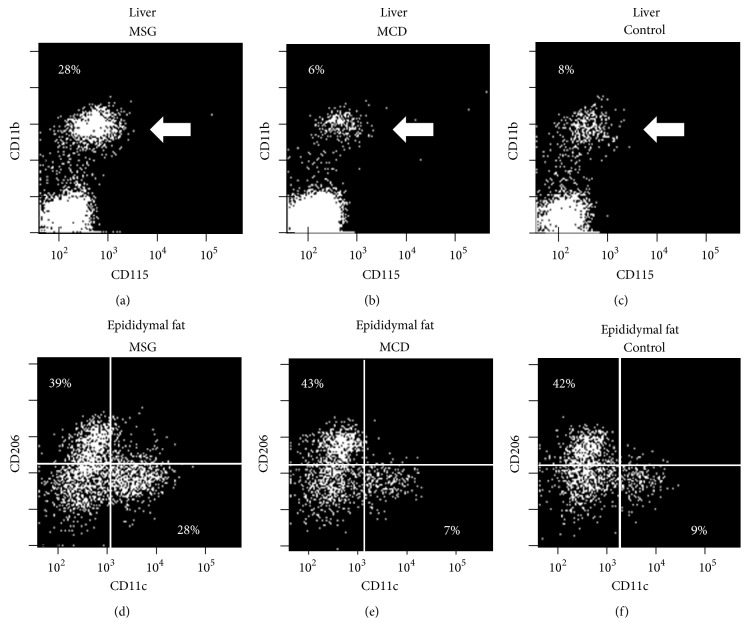
Frequency of monocytes among hepatic mononuclear cells and the frequency of macrophages among the cells in the stromal vascular fraction at 18 weeks. Representative dot plots of the monocyte (arrow), M1 macrophage, and M2 macrophage subpopulations are shown. All samples were triplicated, and data for one representative mouse per group are shown.

After removing macrophages and Kupffer cells, which are strongly positive for F4/80, from the HMNC fraction, mouse hepatic monocytes were identified as a CD11b- and CD115-positive cell population [12]. To assess the number of macrophages that had been present in the epididymal tissue, F4/80-positive cells were collected from the SVF and M1 and M2 macrophages were identified as CD11c-positive/CD206-negative and CD11c-negative/CD206-positive subpopulations, respectively [13]. The proportion of monocytes among the HMNC was 28.3 ± 5.6% in the MSG mice, which was significantly higher than the proportions seen in the MCD (6.2 ± 2.4%) and control mice (8.3 ± 3.8%) (*P* < 0.05) (Figure 4). The proportion of M1 macrophages in the SVF was 28.2 ± 8.7% in the MSG mice, which was significantly higher than the proportions observed in the MCD (7.6 ± 5.6%) and control mice (9.3 ± 6.1%) (*P* < 0.05). The proportion of M2 macrophages in the SVF was comparable in all 3 groups of mice (MSG: 39.4 ± 7.2%, MCD: 43.1 ± 6.8%, and control: 42.2 ± 7.6%, *P* > 0.05).

## 4. Discussion

To create an animal model of NAFLD/NASH, we injected MSG into male C57BL/6J mice, a strain that is widely used as a background for models of human disease such as arteriosclerosis. It has been reported that C57BL/6J mice tend to develop fatty livers more easily than BALB/c mice [14]. Given that C57BL/6J mice are Th1-polarized [15], the Th1 response might play a facilitatory role in the pathogenesis of NASH.

Our study demonstrated that MSG-treated C57BL/6J mice exhibited a similar liver tissue histopathology to that seen in human NAFLD/NASH; increased body weight and visceral fat weight; and elevated blood glucose, serum insulin, total cholesterol, and ALT levels. These results suggest that MSG mice are a useful model of human NAFLD/NASH combined with metabolic syndrome-like characteristics.

It is still unclear why neonatal MSG injections induce metabolic syndrome. Fujimoto et al. reported that ICR-MSG mice that were fed a restrictive diet continued to be obese [16], and Arndt et al. reported that MSG treatment resulted in an increased basal metabolic rate [17]. Therefore, appetite disturbance is not considered to be the main cause of obesity, suggesting that obesity is caused by a reduction in energy metabolism, secondary to brown adipose tissue formation and autonomic nerve dysfunction.

Compared with the MSG mice, the MCD mice displayed lower body weights and reduced amounts of visceral fat as well as decreased blood glucose, serum insulin, and total cholesterol levels, although they also exhibited more marked inflammatory changes in their livers. The pathogenesis of fatty liver involves increased inflow of glucose and free fatty acids into the liver, accelerated biosynthesis of fatty acids, and reductions in the metabolic capacity of mitochondria and the secretion of very low density lipoproteins (VLDL) [18]. In the MCD model, phosphatidylcholine synthesis is inhibited and the secretion of VLDL from the liver is suppressed [19], resulting in the generation of a fatty liver. Thus, individual mechanistic aspects of the MCD model should be evaluated further.

Human NAFLD is associated with elevated monocyte chemotactic protein-1 (MCP-1) levels [20]. In mice, the MCP-1 produced by Kupffer cells has been reported to exert monocyte-specific chemotactic activity and to be involved in inflammation within the liver [21]. It has also been reported that increased numbers of inflammatory M1 macrophages are seen in the adipose tissue of obese mice [22]. In this study, we investigated the frequencies of monocytes in the liver and M1 macrophages in epididymal adipose tissue by flow cytometry. As a result, we detected increased numbers of monocytes and M1 macrophages in the liver and epididymal adipose tissue, respectively, of the MSG mice, which was consistent with previous reports. In recent years, immunometabolism, which involves crosstalk between metabolic and immune systems [23], has drawn attention as an emerging field. MSG mice might represent a useful animal model for studying immunometabolism.

## 5. Conclusion

MSG mice exhibited the characteristic liver histopathology of NASH as well as metabolic syndrome-like features, which suggests that they are a more useful model of human NASH combined with metabolic syndrome than MCD mice.
